# Connor–Davidson Resilience Scale (CD-RISC): Validation in Chinese Physical Education Teachers

**DOI:** 10.21315/mjms-04-2026-225

**Published:** 2026-08-31

**Authors:** Yishuai Wang, Ke Zhou, Yanli Zhou, Yee Cheng Kueh, Garry Kuan

**Affiliations:** 1 Exercise and Sports Science Programme, School of Health Sciences, Health Campus, Universiti Sains Malaysia, 16150 Kubang Kerian, Kelantan, Malaysia; 2 School of Sports Science, Henan University, 475000 Kaifeng, Henan, China; 3 Biostatistics and Research Methodology Unit, School of Medical Sciences, Health Campus, Universiti Sains Malaysia, 16150 Kubang Kerian, Kelantan, Malaysia

**Keywords:** validity, reliability, physical education teacher, resilience, forward-backward translation

## Abstract

**Background:**

Resilience is the ability to bounce back and quickly recover from stressful situations. Resilience is crucial for personal and professional development in today’s rapidly evolving society. This study aims to verify the applicability of the Chinese version of the Connor–Davidson Resilience Scale (CD-RISC-C) to physical education teachers in China through confirmatory factor analysis (CFA).

**Methods:**

A cross-sectional study was conducted in six provinces in China. This study recruited a total of 706 physical education teachers as research participants. Their level of resilience was assessed using the 25-item CD-RISC-C. The scale was translated and culturally adapted from the original English version into Simplified Chinese following rigorous forward–backward translation procedures. CFA was conducted with Mplus 8.0 to examine the psychometric properties of the scale in this sample.

**Results:**

The hypothesised model of the CD-RISC-C consisted of five factors and 25 observed items. In the CFA, the model exhibited a good fit to the data (χ^2^/df = 2.768, RMSEA = 0.046, CFI = 0.956, TLI = 0.950, SRMR = 0.031). Furthermore, the average variance extracted (AVE) values in this study were all above the standard of 0.50, and the composite reliability (CR) value was higher than 0.60, indicating sufficient convergent and discriminant validity. Cronbach’s alpha was 0.967, indicating excellent internal consistency for the CD-RISC-C.

**Conclusion:**

The CD-RISC-C is a valid instrument for assessing resilience among physical education teachers in mainland China.

## Introduction

Resilience is the ability to bounce back and quickly recover from stressful situations (1, 2). This personality characteristic helps individuals manage stress and promotes personal development and growth when facing stressful situations (3). When individuals acquire this ability, they can face crises or significant difficulties by applying strategies and methods, enhancing their self-confidence, thereby demonstrating good coping abilities and achieving positive results (4). Yonezawa et al. (5) emphasised that resilience is not a fixed trait but can be developed and modified. They described it as a dynamic concept that takes shape through interactions among personal strengths, self-efficacy, and the social environments in which people live and work.

Resilience is the ability to bounce back and quickly recover from stressful situations (1, 2). This personality characteristic helps individuals manage stress and promotes personal development and growth when facing stressful situations (3). When individuals acquire this ability, they can face crises or significant difficulties by applying strategies and methods, enhancing their self-confidence, thereby demonstrating good coping abilities and achieving positive results (4). Yonezawa et al. (5) emphasised that resilience is not a fixed trait but can be developed and modified. They described it as a dynamic concept that takes shape through interactions among personal strengths, self-efficacy, and the social environments in which people live and work.

Individuals encounter numerous pressures and challenges from various aspects of life (6). In the workplace, competition is intensifying, and skill requirements are constantly increasing. Individuals must consistently adapt and acquire new knowledge to remain competitive (7). In addition to professional challenges, individuals encounter various pressures in their family and social lives (8). Responsibilities at home, interpersonal relationships, and financial burdens can significantly affect an individual’s psychological well-being (9). Individuals may also need to adapt to new social circles and address interpersonal issues, which requires strong resilience (10). Despite the various challenges that individuals face, resilience can help them cope effectively. Resilience entails adapting to change, managing stress in a constructive manner, maintaining an optimistic attitude, and seeking solutions to problems (11). Therefore, resilience is paramount for individuals’ personal and professional development.

While numerous studies have examined teacher stress and burnout, researchers have increasingly focused on factors that support teachers in not only enduring but also flourishing in educational settings (12). Evidence indicates that teachers with higher levels of resilience are better able to maintain persistence in challenging circumstances while balancing personal needs with student responsibilities (13). There is an ongoing debate over whether resilience is an innate characteristic or a developable quality. Early influential research treated resilience as a fixed attribute that teachers either had or did not have (14). In contrast, contemporary scholars increasingly regard resilience as a quality that can be gradually fostered and enhanced.

Resilience has proven to be very important in helping teachers cope with difficulties and workplace challenges. Teachers with high resilience can handle stressors effectively while still meeting their students’ needs (15). Greater resilience provides teachers with the positive energy needed to cope with demanding work environments (16), including teaching in urban settings (5). Previous research has also shown that teacher effectiveness can be predicted by qualities closely linked to resilience, such as persistence, enthusiasm, and life satisfaction (2).

The Brief Resilience Scale (17) was developed to evaluate one’s capacity to recover and bounce back following stressful experiences. The Academic Resilience Scale (ARS-30) (18) focuses on the process-related dimensions of resilience rather than solely on outcomes, measuring academic resilience by examining students’ specific adaptive cognitive, emotional, and behavioural responses to academic challenges. The Resilience in Midlife Scale (19) is a newly developed instrument for assessing resilience in middle-aged individuals. Additionally, the 28-item Resilience Scale for Adolescents (20) is a reliable and useful tool for studying resilience and associated risk factors in adolescent populations.

The current study employed the Connor–Davidson Resilience Scale (CD-RISC) (21). This scale was first developed in 2003 by American psychologists Kathryn M. Connor and Jonathan R.T. Davidson to address deficiencies in earlier resilience assessment tools. Consisting of five dimensions and 25 items, the CD-RISC offers a comprehensive assessment of individuals’ ability to withstand pressure and recover from setbacks, with an emphasis on self-perception, stress management, and adaptive capacity. The CD-RISC uses a five-point Likert scale (0 to 4 points), with scores ranging from 0 to 100, higher scores indicating greater psychological resilience. Moreover, the CD-RISC is widely used in clinical patient monitoring, assessment of adolescent resilience in education, and psychological screening in high-stress occupations, such as among soldiers and firefighters (22–28). The CD-RISC remains a widely used instrument, with its scientific merit and practicality verified in multiple language versions (25, 29, 30).

The participants selected for this study are physical education (PE) teachers. PE teachers, as a distinct group within the educational system, bear unique responsibilities and face challenges and pressures that significantly differ from those encountered by teachers of other subjects (29). First, the nature of PE teachers’ work requires them to focus not only on students’ academic performance but also on fostering their physical fitness and athletic skills (30). Moreover, the working environment of PE teachers is often challenging and stressful (31). PE teachers may need to teach in different venues and environments, face a variety of weather and climatic conditions (32), and adapt to the characteristics and needs of students of different ages while also dealing with expectations and pressures from parents and schools (33). These external pressures necessitate that PE teachers possess high resilience to cope with unexpected situations and challenges, ensuring stability and effectiveness in their work (34).

Given these occupation-specific stressors, a validated resilience instrument for Chinese PE teachers is needed. Existing Chinese versions of the CD-RISC have used the abbreviated 10-item form and have been tested only in clinical and student samples (35, 36). The 10-item version drops two dimensions from the original scale: control and spiritual influences. Both are directly relevant to the challenges described above. PE teachers must maintain instructional control in outdoor settings where weather, equipment, and safety risks are unpredictable (32). They also rely on personal resolve to cope with the professional marginalisation that comes from working in a subject often viewed as secondary (33). Whether the full 25-item CD-RISC, with its original five-factor structure, adequately captures resilience in this occupational group remains untested. Confirming the scale’s psychometric properties in this population is therefore a necessary step before resilience can be accurately assessed and targeted in intervention research among Chinese PE teachers.

Given that applying psychological characteristic measurement tools in different populations requires not only language translation but also consideration of language characteristics and cultural adaptability, validity and reliability tests must be conducted on the translated scales (37–39). Therefore, the purposes of this study were to translate the CD-RISC into Chinese for use in a Chinese population, examine the reliability and validity of the translated version among Chinese PE teachers, and discuss its adaptability in a Chinese population.

## Methods

### Participants

This study included 706 in-service PE teachers from China. Of the participants, 559 (79.18%) were male, and 147 (20.82%) were female. This gender distribution is consistent with the national profile of Chinese PE teachers. According to the Henan Provincial Department of Education (2023), 71.1% and 28.9% of primary and secondary school PE teachers are male and female, respectively. Although national data are not publicly available, the gender distribution in this study is broadly representative of the target population. All participants were Chinese and demonstrated strong comprehension abilities in reading, speaking, and writing Chinese.

Confirmatory factor analysis (CFA) was used. An ideal sample size is typically 5 to 10 times the number of scale items, according to conventional sample size standards (40, 41). The questionnaire comprised 25 items (the CD-RISC) in this study. Thus, the sample size of 706 was sufficiently large for a CFA study.

### Questionnaire Translation

The original English CD-RISC was translated into Chinese using the following procedure: First, the scale was translated into Chinese, focusing on semantic consistency rather than literal translation. Next, a bilingual Chinese–English speaker back-translated the Chinese version into English. Two expert panels, each comprising 10 members with at least 10 years of experience in sport psychology and education, then reviewed both translations and compared them with the original scale to identify any semantic discrepancies, after which the Chinese version was finalised.

The panels also evaluated the cultural suitability of the scale for Chinese respondents. A pilot test was then conducted among 30 PE teachers to verify the clarity and understandability of the questionnaire. The participants provided feedback on item wording and structure. The pilot results were satisfactory, and no further revisions were required. Both the original English and final Chinese versions are included in the appendices.

### Data Collection

Data were collected from December 2024 to January 2025 across six regions in China: Henan, Jiangsu, Shanghai, Guangdong, Gansu, and Beijing. First, the researcher circulated a poster to all PE teachers in these regions. Thereafter, individuals interested in participating in the study contacted the researcher. Subsequently, the researcher pre-screened these individuals to determine whether they met the inclusion and exclusion criteria before formally inviting them to participate. During this online recruitment process, a total of 706 questionnaires were received. Upon review, all participants met the eligibility criteria. Therefore, all 706 responses were included in the final analysis, as each provided complete answers.

### Measures

#### Demographic Information

To provide a comprehensive understanding of the participants’ profiles, this study first collected and analysed the demographic information of the participants, including gender, age, years of teaching experience, educational background, region, weekly class hours, whether they served as a school sports team coach, and other positions held at school.

#### Connor–Davidson Resilience Scale

Connor and Davidson (2003) constructed the CD-RISC, which includes 25 items scored on a 5-point Likert scale: 0 for not true at all, 1 for rarely true, 2 for sometimes true, 3 for often true, and 4 for true nearly all of the time. Respondents rated each statement according to their feelings over the previous month. The total scores range from 0 to 100, with higher values indicating greater resilience. The scale has been verified in both general and clinical groups, demonstrating acceptable psychometric properties, including strong internal consistency and test–retest reliability.

### Ethics Approval

This study was carried out in line with the Declaration of Helsinki and approved by the Human Research Ethics Committee of Universiti Sains Malaysia (USM/JEPeM/KK/23090691). Before the investigation, all participants received a detailed research information sheet. By voluntarily completing and returning the CD-RISC questionnaires, participants provided implied consent to participate. They were guaranteed that all their responses would be kept confidential, and they were informed that they could withdraw from the research at any time without any consequences. The questionnaire contained no information that could identify individual participants.

### Statistical Analysis

Online questionnaire data were collected directly using Questionnaire Star and analysed using Mplus 8.0. Standard data-screening procedures were performed before formal analysis (42), and tests of normality and linearity were conducted. After screening, valid items were retained, and the internal consistency of the full scale and its subscales was evaluated using Cronbach’s alpha. All factors exceeded the 0.70 threshold for acceptable reliability, and composite scores were created by averaging indicators within each factor.

CFA rather than exploratory factor analysis (EFA) was employed because the CD-RISC has a well-established five-factor theoretical structure that has been validated across multiple cultures and populations (25, 29, 30). The present study aimed to confirm this established factor structure in a new population (Chinese PE teachers) rather than explore alternative structures.

#### CFA

CFA was performed to evaluate the psychometric properties of the measurement tools. All variables were included in a single CFA model to assess correlations among constructs and convergent and discriminant validity (42). Convergent validity was assessed using factor loadings, composite reliability (CR), and average variance extracted (AVE). Factor loadings with *t*-values greater than 1.96 were significant at α = 0.05 (43). CR values above 0.70 and AVE values of at least 0.50 were regarded as satisfactory (27, 44). Discriminant validity was assessed by examining the correlations among the variables.

#### Model Fit Indices

Fit statistics were applied in all latent variable analyses to evaluate the model–data fit (45). A satisfactory fit supports the proposed structural relationships, whereas a poor fit indicates model misspecification (46). Multiple fit indices were used in this study: χ^2^/df, RMSEA, GFI, NFI, RFI, CFI, and IFI. A χ^2^/df ratio between 1 and 5 is generally acceptable, with a ratio of 1 to 3 indicating an excellent fit. RMSEA ≤ 0.10 indicates an acceptable model, and values below 0.05 indicate an excellent fit (47). GFI, AGFI, IFI, NFI, and CFI range from 0 to 1, with values closer to 1 indicating better model fit (48).

## Results

### Descriptive Statistics

Table 1 presents the demographic characteristics of the 706 study participants. Among them, 559 were male (79.18%), and 147 were female (20.82%). Most respondents were aged 25 to 45 years, accounting for 575 participants (81.45%). Regarding teaching experience, the largest groups were those with 3 to 8 years of service (212 individuals, 30.03%) and 9 to 15 years (179 individuals, 25.35%).

The educational background was mainly undergraduate, with 558 participants holding bachelor’s degree (79.03%). The majority of participants (*n* = 623) were from urban areas (88.24%), with the remaining 83 from rural areas (11.76%). Most PE teachers taught 11 to 15 weekly lessons (*n* = 248, 35.13%). Half of the PE teachers served as school sports team coaches (*n* = 359, 50.97%), and 214 participants (30.31%) held other positions at the school.

### CFA

In this study, resilience was measured using the five-factor Chinese version of the CD-RISC. As shown in Figure 1, the standardised factor loadings of the items ranged from 0.731 to 0.816.

The initial CFA results indicated that the model fit indices were within the acceptable range (Table 2). Given that all items had factor loadings above 0.4, no items were deleted.

Table 3 displays the CR and AVE values of the CD-RISC-C. For Factor 1, the CR was 0.848, and the AVE was 0.583. Factor 2 yielded a CR of 0.809 and an AVE of 0.585. Factor 3 achieved a CR of 0.919 and an AVE of 0.619. The CR and AVE for Factor 4 were 0.908 and 0.584, respectively, while Factor 5 recorded a CR of 0.823 and an AVE of 0.608.

### Internal Consistency

The Cronbach’s alpha value for the CD-RISC-C was 0.967 (Table 4). Moreover, the McDonald’s Omega value for this model was 0.961, indicating excellent internal consistency across the CD-RISC-C.

### Test–Retest Reliability

We performed a test–retest reliability analysis with 60 participants over a two-week interval to evaluate the scale’s stability. The average score on the CD-RISC-C changed slightly from 78.5 (Day 1, SD = 24.1) to 79.3 (Day 14, SD = 24.6). The intraclass correlation coefficient was 0.860 (95% CI = 0.825, 0.931; *P* < 0.001), which supported acceptable stability of the instrument.

## Discussion

This study evaluated the reliability and validity of the Chinese version of the CD-RISC-C using CFA. The study subjects were Chinese secondary school PE teachers. The assessment of the development, validity, and reliability of the CD-RISC-C is a key step in conducting research on work motivation in the Chinese population. Based on previous research, the original English version of the CD-RISC has demonstrated cross-time stability and good reliability and validity index values (22, 23). The scale has been translated into multiple languages, making it widely applicable for measuring the psychological characteristics of different research groups (24, 25, 28). Although previous studies have examined the reliability and validity of the CD-RISC, discussions and research on resilience in the Chinese context have focused on adolescents and healthcare workers (35, 36), with no resilience measurement tool for teachers, especially PE teachers. This gap was filled by testing the CD-RISC-C on Chinese PE teachers. This tool is an important means of measuring the psychological characteristics of Chinese PE teachers.

In this study, CFA was employed to examine the construct validity and psychometric properties of the Chinese version of the CD-RISC-C, as this method assesses how well the scale items correspond to the theoretical dimensions they are intended to measure (49–52). Previous validation studies on other translated editions of the CD-RISC have yielded acceptable results using similar procedures (23, 24). This research focused on confirming rather than exploring the factorial structure of the Chinese CD-RISC-C, which is why CFA was adopted over EFA. Based on the scale items and the reliability and validity outcomes, the five-factor structure demonstrated satisfactory model fit and was consistent with both the original scale and prior research on the Chinese version. Regarding the items of the scale, only the Chinese version was translated in this study, without any cuts or adjustments, and it is consistent with the original version (27, 21). Overall, the analysis showed that the CD-RISC-C is an excellent psychometric measure with high factor loadings and exhibited good model–data fit. This result is reassuring for studies that use the CD-RISC-C to measure resilience levels.

After confirming the factor structure, its reliability and validity were further evaluated. Reliability refers to the stability and consistency of a measurement tool, while validity refers to whether the tool accurately measures the concept or phenomenon being assessed (53, 54). First, this study examined the CR and discriminant validity of the CD-RISC-C. The AVE values in this study were all above the standard of 0.50, and the CR was above 0.60, both of which met the ideal range. Second, the internal consistency and test–retest reliability were excellent. These results indicate that the CD-RISC-C demonstrated good validity and reliability in this study’s sample. This finding is consistent with the high consistency reported in numerous previous studies that validated the CD-RISC (25, 26, 28). Overall, this result is encouraging for research on measuring resilience using CD-RISC-C.

The importance of resilience in stress management among adults has been widely recognised (6). Resilience is a personality trait or stable ability. When individuals encounter stressful events in life, this trait or ability can help ensure that they cope with the pressure and promote their growth and development (55). This ability not only plays an essential role in the development and success of individuals but also profoundly influences the progress and development of society as a whole (56). Therefore, validating Chinese versions of resilience measurement tools is crucial. When adapting resilience scales to the Chinese context, it is essential to first recognise the influence of cultural background on resilience (57). Chinese culture, social systems, and work characteristics may differ from those in the West. Individuals from different cultural backgrounds have different attitudes toward work, values, and behavioural habits (58). Therefore, the resilience measurement tools developed in English may not accurately reflect the resilience of individuals in the Chinese cultural context and must be adjusted to local and cultural contexts.

Research shows that resilient teachers can manage stress according to the needs of their students (2). Resilience has proven to be a key factor in understanding how some teachers respond to difficulties, while others succumb to challenges in their work lives (59). As professionals engaged in PE, PE teachers must have good resilience to cope with various work-related challenges and pressures (60). Accordingly, choosing PE teachers as the research participants is reasonable and appropriate. The working environment of PE teachers is often challenging and stressful. PE teachers may need to teach in different venues and environments, face a variety of weather and climatic conditions, and adapt to the characteristics and needs of students of different ages while also dealing with expectations and pressures from parents and schools (61).

However, further research is needed to determine the reproducibility of CD-RISC-C in other populations, such as teachers of other subjects, individuals of different ages and educational levels, and even more diverse groups in professions other than teaching. Researchers should also apply longitudinal studies to examine the changes and stability of CD-RISC-C over time. Longitudinal studies can provide more information than cross-sectional studies, measuring variability or constancy and examining potential causal relationships.

We acknowledge some limitations in this study, as our population is restricted to PE teachers. PE teachers are a small group in China and a minority among all teachers, which may limit the generalisability of these findings to other populations. The substantial gender imbalance (79.18% male), while representative of Chinese PE teachers, limited our ability to test measurement invariance across gender. The small female subsample (*n* = 147) did not meet the minimum requirements for stable multi-group CFA. Future studies should oversample female PE teachers to examine whether the five-factor structure holds equivalently across gender groups. Although our study had some limitations, particularly regarding population selection, this does not mean that we could not gain valuable insights or draw important conclusions.

The sample size of this study is limited, indicating that it cannot cover the entire country. Only a few provinces in China were selected for investigation. Although we have attempted to make this study as extensive as possible, we are fully aware of its limitations. In addition, our study used self-reported data collected via an online survey. Self-reported participant or response bias may reduce the accuracy of the data (62). Moreover, participants may be influenced by social desirability bias and answer questions in ways designed to make themselves look good (63). Online surveys can be anonymous, and participants do not provide their names; thus, they know that the researchers cannot identify them, reducing the chances of responding in a social desirable manner. However, the results obtained in this study are satisfactory, and the reliability and validity tests are excellent.

### Applicable Remarks

The manuscript investigates the psychometric properties of the CD-RISC-C through a mixed-methods approach, encompassing both quantitative and qualitative analyses. We conducted the study with a sample of 706 PE teachers in China, utilising Mplus 8.0 software for CFA. Our results indicated that the CD-RISC-C consistently demonstrates strong reliability and validity, comparable to the original CD-RISC scale.

Additionally, this study explores the broader impact of resilience on the mental health and career development of Chinese PE teachers, thereby contributing to the understanding of how cultural contexts influence psychosocial assessments.

The findings from this study underscore the need for culturally relevant assessment tools that facilitate further research into coping strategies and mental health outcomes in China.

## Conclusion

The final measurement model of the CD-RISC-C in this study included all 25 items, and each item was retained as suitable for the study sample. Following model validation, the scale demonstrated satisfactory reliability and validity, making it appropriate for use in the Chinese cultural context. The five-factor CD-RISC-C can be applied in future studies examining resilience-related psychological characteristics among Chinese-speaking populations.

## Figures and Tables

**Figure 1:**
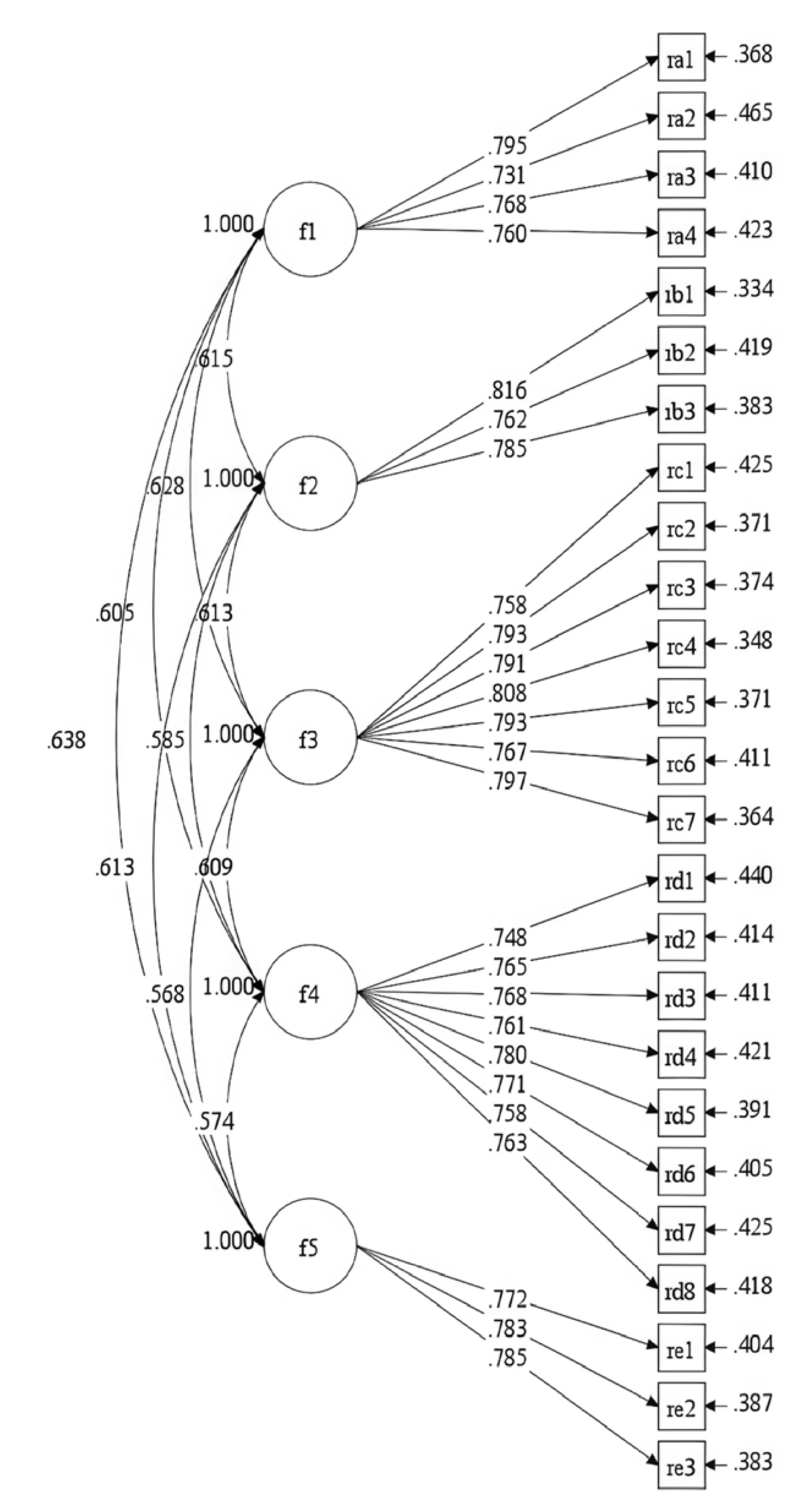
Initial model of resilience with five factors. f1 reflects the notion of personal competence, high standards, and tenacity; f2 corresponds to trust in one’s instincts, tolerance of negative affect, and the strengthening effects of stress; f3 relates to positive acceptance of change and secure relationships; f4 relates to control; and f5 represents spiritual influences.

**Table 1:** Demographic characteristics of participants (*n* = 706).

		*n*	Percentage
Gender	Male	559	79.18
	Female	147	20.82

Age	< 25	31	4.39
	25 to 35	248	35.13
	36 to 45	327	46.32
	> 46	100	14.16

Age of teaching	< 3	145	20.54
	3 to 8	212	30.03
	9 to 15	179	25.35
	16 to 20	100	14.16
	> 20	70	9.92

Educational background	Junior college education	40	5.67
	Undergraduate	558	79.03
	Postgraduate	108	15.30

Region	City	233	33.00
	Country	390	55.24
	Countryside	83	11.75

Weekly class hours	< 10	61	8.64
	10 to 15	103	14.59
	16 to 20	161	22.80
	21 to 25	248	35.13
	> 25	133	18.84

School sports team coach	Yes	359	50.97
	No	347	49.03

Held other positions in the school	Yes	492	69.69
	No	214	30.31

**Table 2:** GFI for the CD-RISC-C with five factors (cut-off and actual values).

Model fitting index	*χ* ^2^	df	*χ*^2^/df	RMSEA	CFI	TLI	SRMR
Cut-off value			< 5	< 0.08	> 0.90	> 0.90	< 0.08
Actual value	733.750	265	2.768	0.046	0.956	0.950	0.031

χ^2^ = Chi-square; df = degrees of freedom; RMSEA = root mean square error of approximation; CFI = comparative fit index; TLI = Tucker–Lewis Index; SRMR = standardised root mean square residual.

**Table 3:** Construct validity for the measurement models.

	CR	AVE
**CD-RISC includes five factors**		

Factor 1	0.848	0.583
Factor 2	0.809	0.585
Factor 3	0.919	0.619
Factor 4	0.908	0.584
Factor 5	0.823	0.608

Factor 1 reflects the notion of personal competence, high standards, and tenacity; Factor 2 corresponds to trust in one’s instincts, tolerance of negative affect, and the strengthening effects of stress; Factor 3 relates to positive acceptance of change and secure relationships; Factor 4 relates to control; and Factor 5 represents spiritual influences.

**Table 4:** Summary of Cronbach’s alpha and McDonald’s Omega values for the CD-RISC-C.

Variables	Cronbach’s alpha	McDonald’s Omega
CD-RISC-C	0.967	0.961
